# Should motivational interviewing training be mandatory for medical students?

**DOI:** 10.3402/meo.v21.31272

**Published:** 2016-02-26

**Authors:** Lara Shemtob

**Affiliations:** UCL Medical School, School of Life and Medical Sciences, University College London, London, United Kingdom

Many medical students feel disillusioned when they step into the clinical environment. Years of preclinical study leaves us enthused about using our knowledge to make a difference to people's lives. We are met with the reality that patients find it difficult to be compliant with the evidence-based medication regimes and lifestyle changes recommended to them. Clinicians have the same frustrations. Many patients are not ready for change, do not comply with treatment, and do not engage with the multidisciplinary help that is on offer to them. We have all witnessed conversations where the doctor and patient are pushing different agendas, the professional relationship is strained, and ultimately no one's objectives are met.

One evidence-based approach to encourage a patient along the cycle of change (1) is motivational interviewing (MI). Traditionally used in settings like drug addiction services, this technique is being successfully applied to many other areas of medicine (2). As part of the UCL Patient Centred Pathway, fifth year medical students have had the opportunity to study and practice MI skills. By following a chronically ill patient through time in a longitudinal pathway, we have been able to use these techniques to try to encourage the patient to embrace help to change their lives for the better. The link between learning MI skills and learning how to support patients in self-managing chronic illness is recognised (3). Longitudinal patient pathways are a valuable means of learning, where through time and multiple meetings a strong student patient rapport can be established (4). Integrating MI skills into a longitudinal pathway is a concept with potential (5), with possible benefits for both patients and students.

The programme I took part in was met with a mixed response from students. Some felt following acronyms and flowcharts to structure the consultation retracted from a valuable natural student–patient relationship that built over time. Others reported feeling empowered by these skills, making more progress with their patients than they thought possible at the start of the project. It helped me understand a patient-centred approach to medicine. I used to feel frustrated and disappointed witnessing consultations where patients failed to engage with interventions and even questioned the applicability of patient autonomy to a few situations. Now I realise change is more powerful when it comes from the patient themselves, and the skill lies in getting the patient to seek change, rather than enforcing it upon them. This has significantly changed my outlook to patient communication. There is a growing evidence base for the advantages of training medical students in MI skills (6, 7). How these skills are best delivered to clinicians of the future is being internationally researched and refined with respect to post graduate (8), and undergraduate curricula across healthcare professions (9, 10). If we were to integrate MI skills into undergraduate medical education, clinical examinations, and professional training, it would become second nature in all consultations. Every patient interaction could have a greater chance of helping the patient engage with their situation, bringing them closer to compliance, lifestyle changes, and better health.

There are a growing number of chronic illness patients managing their conditions themselves in the community with varying degrees of professional involvement. Exploiting the patient-centred approach to produce a patient more engaged with dealing with their own illness reduces the burden on the health system (11) as well as improving individual outcomes (12, 13). The accumulation of multiple chronic comorbidities in complex patients is something each new generation of doctors has to deal with in an even more stretched health system. It is key to the future of any health service for clinicians to learn how to engage our patients to take on more responsibility themselves.

The balance between a patient's best interests and their right to autonomy is something medical students become familiar with during their clinical education. Movement away from a paternalistic to a patient-centred doctor–patient relationship (14) emphasises that it is the responsibility of the clinician to provide the opportunity for change, not to enforce change. Patient engagement is key to successful medicine, across specialities. Proper training and experience in MI will make medical students more useful in the health service from the start of our careers. As clinicians of the future, I believe we ought to have these skills built into our practice from the outset.

*Lara Shemtob* UCL Medical School
 School of Life and Medical Sciences
 University College London
 London, United Kingdom
 Email: lara.shemtob.11@ucl.ac.uk
